# Sensitization of human cancer cells to gemcitabine by the Chk1 inhibitor MK-8776: cell cycle perturbation and impact of administration schedule *in vitro* and *in vivo*

**DOI:** 10.1186/1471-2407-13-604

**Published:** 2013-12-21

**Authors:** Ryan Montano, Ruth Thompson, Injae Chung, Huagang Hou, Nadeem Khan, Alan Eastman

**Affiliations:** 1 Department of Pharmacology and Toxicology, Geisel School of Medicine at Dartmouth, Lebanon, NH USA; 2 Duksung Women’s University, Seoul, Korea; 3 Department of Radiology, Geisel School of Medicine at Dartmouth, Lebanon, NH USA; 4 Norris Cotton Cancer Center, Geisel School of Medicine at Dartmouth, Rubin Building Level 6, Lebanon, NH USA

**Keywords:** Chk1, Gemcitabine, MK-8776, Drug combinations, Pancreas cancer xenografts, Homologous recombination, Cell cycle perturbation

## Abstract

**Background:**

Chk1 inhibitors have emerged as promising anticancer therapeutic agents particularly when combined with antimetabolites such as gemcitabine, cytarabine or hydroxyurea. Here, we address the importance of appropriate drug scheduling when gemcitabine is combined with the Chk1 inhibitor MK-8776, and the mechanisms involved in the schedule dependence.

**Methods:**

Growth inhibition induced by gemcitabine plus MK-8776 was assessed across multiple cancer cell lines. Experiments used clinically relevant “bolus” administration of both drugs rather than continuous drug exposures. We assessed the effect of different treatment schedules on cell cycle perturbation and tumor cell growth in vitro and in xenograft tumor models.

**Results:**

MK-8776 induced an average 7-fold sensitization to gemcitabine in 16 cancer cell lines. The time of MK-8776 administration significantly affected the response of tumor cells to gemcitabine. Although gemcitabine induced rapid cell cycle arrest, the stalled replication forks were not initially dependent on Chk1 for stability. By 18 h, RAD51 was loaded onto DNA indicative of homologous recombination. Inhibition of Chk1 at 18 h rapidly dissociated RAD51 leading to the collapse of replication forks and cell death. Addition of MK-8776 from 18–24 h after a 6-h incubation with gemcitabine induced much greater sensitization than if the two drugs were incubated concurrently for 6 h. The ability of this short incubation with MK-8776 to sensitize cells is critical because of the short half-life of MK-8776 in patients’ plasma. Cell cycle perturbation was also assessed in human pancreas tumor xenografts in mice. There was a dramatic accumulation of cells in S/G_2_ phase 18 h after gemcitabine administration, but cells had started to recover by 42 h. Administration of MK-8776 18 h after gemcitabine caused significantly delayed tumor growth compared to either drug alone, or when the two drugs were administered with only a 30 min interval.

**Conclusions:**

There are two reasons why delayed addition of MK-8776 enhances sensitivity to gemcitabine: first, there is an increased number of cells arrested in S phase; and second, the arrested cells have adequate time to initiate recombination and thereby become Chk1 dependent. These results have important implications for the design of clinical trials using this drug combination.

## Background

DNA damage activates cell cycle checkpoints that arrest cell cycle progression and thereby provide time for repair and recovery. This has led to the development of checkpoint inhibitors as adjuvants to DNA damaging agents with the anticipation that they will enhance therapeutic activity. Chk1 is the primary checkpoint protein against which many small molecule inhibitors have been developed [1-3]. Chk1 is activated when the kinases ATM and/or ATR detect double-strand breaks or large single-strand regions of DNA, respectively. Once activated, Chk1 phosphorylates and inactivates CDC25 phosphatases that are required for CDK activation and cell cycle progression. Inhibition of Chk1 results in premature activation of CDC25 phosphatases and CDK1/2, and progression through the cell cycle before adequate repair has occurred. Increased DNA damage occurs as cells progress through S phase with a damaged template, followed by lethal mitosis once they have reached the G2 phase [4].

Antimetabolites such as gemcitabine and hydroxyurea inhibit ribonucleotide reductase, thereby rapidly depleting deoxyribonucleotide pools and stalling replication fork progression. These agents do not directly induce DNA breaks, and arrest occurs without the need for Chk1 activation. However, Chk1 stabilizes the stalled replication forks and, when inhibited, the replication forks collapse thus producing DNA double-strand breaks [5]. Hence, there is a significant difference in the outcome of Chk1 inhibition depending on the type of DNA damage that occurs; in the latter case, new lethal events occur where no DNA damage existed previously. Consequently, we have found that Chk1 inhibition can induce a far more dramatic sensitization to antimetabolites that induce this replication arrest compared to other DNA damaging agents that activate Chk1 through the DNA damage-induced checkpoint [6].

Gemcitabine is a deoxynucleoside analogue that is metabolized to a deoxynucleotide triphosphate, a precursor for incorporation into DNA, and to a deoxynucleotide diphosphate that irreversibly inhibits ribonucleotide reductase. As a consequence, low concentrations of gemcitabine rapidly deplete deoxyribonucleotide pools, inhibit DNA synthesis and induce a long S phase arrest. Here we focus on the combination of gemcitabine with the Chk1 inhibitor MK-8776 [7]. We report the efficacy of this combination in cell lines from many different cancers. We also report that the time of addition of MK-8776 can significantly impact the response of tumor cells to gemcitabine both *in vitro* and in xenograft tumor models. The schedule dependence is critical because of the relatively short half-life of MK-8776 in patients’ plasma [8]. These results have important implications for the design of clinical trials of this combination.

## Methods

### Materials

Gemcitabine was obtained from Eli Lilly, Indianapolis, IN. MK-8776 (previously known as SCH 900776) was provided by Merck, Kenilworth, NJ and dissolved in dimethylsulfoxide [7]. The majority of cell lines are part of the NCI60 panel and were obtained from the Developmental Therapeutics Program, National Cancer Institute, Bethesda and maintained in RPMI1640 medium plus serum and antibiotics [9]. Other cell lines were obtained from American Type Culture Collection (Manassas, VA). All lines were used within three months of thawing from frozen stocks. No further reconfirmation of their identity was performed.

### Cell analysis

Cell cycle analysis was performed by flow cytometry as described previously [10]. For cell growth assays, cells were seeded at low density (500–1000 cells) in 96-well plates and then incubated with drugs for various schedules usually for 24 h (8 wells per concentration). Following treatment, cells were washed and grown in fresh media for 6–7 days at 37°C. Prior to attaining confluence, cells were washed, lysed, and stained with Hoechst 33258, as previously described [11]. Fluorescence was read on a microplate spectrofluorometer (Spectramax M2). Results are expressed as the concentration of drug that inhibited growth by 50% (IC_50_).

### Immunoblotting

Cells were harvested and analyzed as previously detailed [12] with the following antibodies: phosphoserine-345-Chk1, phosphoserine-296-Chk1, DNA-PK and γH2AX (Cell Signaling); Chk1 (Santa Cruz Biotechnology); phospho-2056-DNA-PK (Abcam); and actin (Sigma).

### Immunofluorescence

Cells were cultured on glass coverslips, incubated with gemcitabine and/or MK-8776, and fixed with 3% paraformaldehyde (20 min at room temperature). The cells were then washed 4 × 15 min in PBS-T (PBS containing 0.15% BSA and 0.1% Triton-X-100). Slides were then incubated with 200 ng/ml anti-Rad51 (Santa-Cruz) overnight, washed in PBS-T and incubated with Alexa-555 conjugated goat anti-rabbit IgG (Invitrogen) at 1:1000 dilution for 1 h. DAPI (1 μg/mL) was added to the final wash and the coverslips were mounted using Prolong Gold Antifade (Invitrogen). Confocal images were acquired using a Zeiss LSM 510 microscope.

### Analysis of tumor xenografts

All animal procedures were performed in strict accordance with the NIH Guide for the Care and Use of Laboratory Animals and approved by the Institutional Animal Care and Use Committee at Dartmouth. To generate tumor xenografts, 2 × 10^6^ AsPC-1 or MiaPaCa-2 pancreas cancer cells were injected into the flanks of athymic nu/nu mice. Drug treatments began after the tumors had reached 100 mm^3^. Gemcitabine was administered at 150 mg/kg i.p. in phosphate buffered saline while MK-8776 was administered at 50 mg/kg i.p. in (2-hydroxypropyl) β-cyclodextrin, 45% w/v solution in water (Sigma). These doses were selected based on a prior publication with these agents [7]. The schedules of administration varied with experiment and are described in the results. Tumors were measured with calipers in two dimensions and volume calculated based on the equation volume = π/6 × length × width^2^. The comparisons between groups at each time point were made using a student’s t test for unpaired samples. The tests were two-sided and a change with a p-value <0.05 was considered statistically significant.

Some tumors were harvested, fixed in formalin, and serial sections were stained with anti-Ki67 (Vector Laboratories) and anti-geminin (Santa-Cruz) in the Research Pathology Shared Resource. For each tumor, at least 2 fields from each of 2 sections were photographed, each field representing about 1000 cells; 2–4 individual tumors were scored at each time point. The number of cells positive for geminin was expressed as a percentage of those positive for Ki67.

## Results

### Impact of MK-8776 on gemcitabine-induced cytotoxicity

We previously analyzed MDA-MB-231 and MCF10A cell lines for sensitivity to gemcitabine alone or when combined with MK-8776 [6]. This analysis has now been expanded to a large panel of cell lines (Table 1). In this assay, cells were incubated with drugs for 24 h, and cell growth was then assessed after an additional 6–7 days. The results are expressed as the IC50 for gemcitabine alone or when incubated with low (200 nmol/L) or high (2 μmol/L) MK-8776; these concentrations were selected based on our prior experience showing differential sensitivity of cell lines to this drug [6]. The cells exhibit a wide range of sensitivity to gemcitabine alone (3 – 83 nmol/L), but concurrent incubation with 2 μmol/L MK-8776 resulted in an IC50 of <6.5 nmol/L for all the cell lines. This reflected a 4–66 fold (median 7) sensitization to gemcitabine. We previously noted that some cell lines are particularly sensitive to MK-8776 alone; these included U2OS, A498 and TK10 [6]. Our expanded screen has now identified AsPC-1 as sensitive to MK-8776 (IC50 0.5 μmol/L following a 24 h incubation and assayed after 7 days). Most of the other cell lines tolerated 10 μmol/L MK-8776 for 24 h. For the sensitive cell lines, it was not possible to determine an IC50 for gemcitabine in combination with 2 μmol/L MK-8776. However in these cell lines sensitization was still observed when combined with 200 nmol/L MK-8776. TK10 cells are an exception in this regard as they are very sensitive to gemcitabine alone so were not sensitized further.

**Table 1:** Sensitivity of cell lines to gemcitabine alone or in combination with MK-8776

**A. Gemcitabine and MK-8776 0–24 h**			
**Cell line**	**IC**_**50 **_**(nmol/L)**		
	**Gem alone**	**Gem + 200 nmol/L MK-8776**	**Gem + 2 μmol/L MK-8776**
U251	36.6 ± 8.8	15 ± 5.5 (2.4)	6.5 ± 1.5 (5.6)
HCT115	25 ± 5	13.8 ± 1.3 (1.8)	5.1 ± 1.1 (4.9)
SW620	83.3 ± 16	30 ± 0 (2.8)	4.8 ± 0.9 (17.4)
IGROV-1	25 ± 5	5.2 ± 1.1 (4.8)	3.5 ±1.5 (7.1)
HCT116	13.8 ± 1.3	5.5 ± 0.5 (2.5)	3.3 ± 0.3 (4.2)
MCF10A	13 ± 6.1	5.2 ± 2.4 (2.5)	2.8 ± 1.1 (4.6)
MiaPaCa-2	26.5 ± 4.3	4.2 ± 0.8 (6.3)	2.2 ± 1.2 (12.0)
MDA-MB-231	18.5 ± 7.1	4.4 ± 0.81 (4.2)	1.5 ± 0.44 (12.3)
HCC2998	15 ± 5	3.8 ± 1.3 (3.9)	1.5 ± 0.4 (10.0)
U87	7.5 ± 1.3	2.7 ± 0.4 (2.8)	1.5 ± 0.3 (5.0)
MDA-MB-435	8.6 ± 3.2	1.5 ± 1.5 (5.7)	0.4 ± 0 (21.5)
SNB19	10 ± 3.5	1.3 ± 0.08 (7.7)	0.15 ± 0.03 (66.7)
U20S	32.5 ± 2.5	10.5 ± 4.5 (3.1)	DEAD
A498	22.5 ± 2.5	3.5 ± 1.5 (6.4)	DEAD
TK10	3.4 ± 1.6	2.8 ± 1.3 (1.2)	DEAD
AsPC-1	14 ± 1	3.3 ± 0.2 (4.2)	DEAD
**B. Gemcitabine 0–24 h; MK-8776 18–24 h**			
U251	36.6 ± 8.8	32.5 ± 2.5 (1.1)	12.5 ± 2.5 (2.9)
MDA-MB-231	18.5 ± 7.1	12.5 ± 5.5 (1.5)	4.6 ± 0.8 (4.0)
U87	7.5 ± 1.3	5 ± 0.6 (1.5)	2.8 ± 0.16 (2.7)
MDA-MB-435	8.6 ± 3.2	8 ± 2 (1.1)	0.77 ± .73 (11.2)
AsPC-1	14 ± 1	7.8 ± 2.2 (1.8)	1.7 ± 0.23 (8.2)
**C. Gemcitabine 0–6 h; MK8776 18–24 h**			
U251	250 ± 51	187 ± 38 (1.3)	113 ± 18.5 (2.2)
MiaPaCa-2	175 ± 25	90 ± 35 (1.9)	39 ± 1 (4.5)
MDA-MB-231	60.5 ± 10.3	35 ± 9.6 (1.7)	19.2 ± 2.3 (3.2)
U87	12.6 ± 3.9	9 ± 2.1 (1.4)	5.3 ± 0.9 (2.4)
MDA-MB-435	41.6 ± 19.7	22.5 ± 7.5 (1.8)	5.5 ± 2.5 (7.6)
SNB19	75 ± 0	35 ± 5 (2.1)	16.5 ± 1.5 (4.5)
AsPC-1	115 ± 11.9	53.3 ± 6.7 (2.2)	12 ± 1.7 (9.6)

Following treatment as indicated, drugs were removed and cells were incubated for an additional 5–7 days and the IC50 for gemcitabine determined.

Values reflect mean +/− SEM with n = 2–5; parenthesis = fold sensitization.

“DEAD” = MK-8776 alone markedly suppressed growth so no cumulative cytotoxicity calculable.

### Cell cycle perturbation induced by gemcitabine and MK-8776

**Figure 1:**
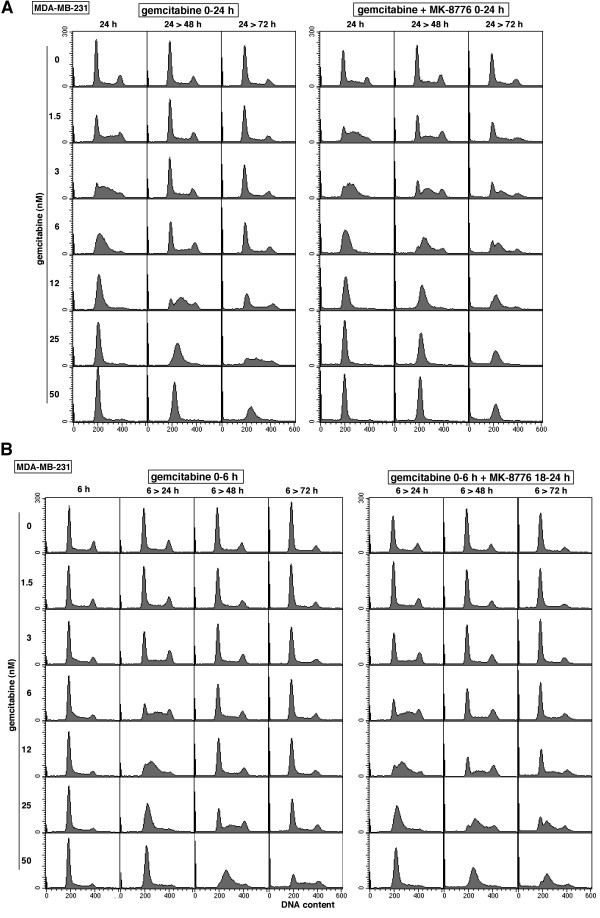
**Impact of gemcitabine and MK-8776 on cell cycle perturbation of MDA-MB-231 cells. A**. Cells were incubated with 0 – 50 nmol/L gemcitabine for 24 h without (left) or with (right) 1 μmol/L MK-8776. The drugs were then removed and cells incubated for an additional 24 or 48 h. Cells were then analyzed for DNA content by flow cytometry. **B**. Similar to A except cells were incubated with gemcitabine for only the first 6 h, while MK-8776 was added only from 18–24 h.

We next determined whether the concentration of gemcitabine that inhibited growth correlated with S phase arrest (Figure 1A). The breast tumor cell line MDA-MB-231 was incubated with gemcitabine for 24 h and the extent of cell cycle perturbation was assessed over the following 48 h. Cells incubated with 3–6 nmol/L gemcitabine accumulated in mid to early S phase by 24 h and appeared to recover completely within 24 h of drug removal. Cells incubated with 12 nmol/L gemcitabine arrested early in S phase at 24 h, progressed further into S phase 24 h after drug removal, and had almost completely recovered by 48 h. This pattern can be compared to the IC_50_ of 18 nmol/L in this cell line (Table 1). In contrast, cells incubated with 50 nmol/L gemcitabine showed very little recovery, and a sub-G1 population began to appear 48 h after release.

We performed parallel experiments to assess cell cycle perturbation when gemcitabine was combined with MK-8776 (Figure 1A). When cells were co-incubated with this combination for 24 h, there was little difference in the cell cycle distribution compared to treatment with gemcitabine alone except at the lowest concentration (1.5 nmol/L) at which there was a further increase in S phase cells. These cell cycle perturbations are important as they relate to the mechanism of action of gemcitabine. Gemcitabine both inhibits ribonucleotide reductase and is incorporated into DNA to cause strand termination. In the face of DNA damage, Chk1 inhibition normally abrogates S phase arrest and drives cells into G2 as we previously observed with the topoisomerase I inhibitor SN38 [13]. However, inhibition of Chk1 did not abrogate S phase arrest induced by gemcitabine. This is explained by the inhibition of ribonucleotide reductase; as there are no deoxyribonucleotides that can be incorporated into DNA, inhibition of Chk1 can not force cells to progress through S phase. This suggests that the majority of the effect of gemcitabine in these experiments is due to inhibition of ribonucleotide reductase.

The most notable impact of MK-8776 occurs following removal of the drugs. After an additional 48 h, there is very little recovery except at the lowest concentration of gemcitabine. The partial recovery at 3 nmol/L gemcitabine is consistent with the IC50 for gemcitabine when combined with 2 μmol/L MK-8776 (Table 1). Hence, this enhanced cytotoxicity occurs at concentrations of gemcitabine that transiently perturb the cell cycle and is therefore consistent with disruption of replication fork progression as discussed further below. At higher concentrations of gemcitabine, there is only slight movement of the cells in S phase and an increasing proportion of cells appear with sub-G1 DNA content. These results are consistent with the cytotoxicity data showing the marked sensitization that occurs when MK-8776 is added to gemcitabine.

### Activation of the DNA damage response by gemcitabine and MK-8776

**Figure 2:**
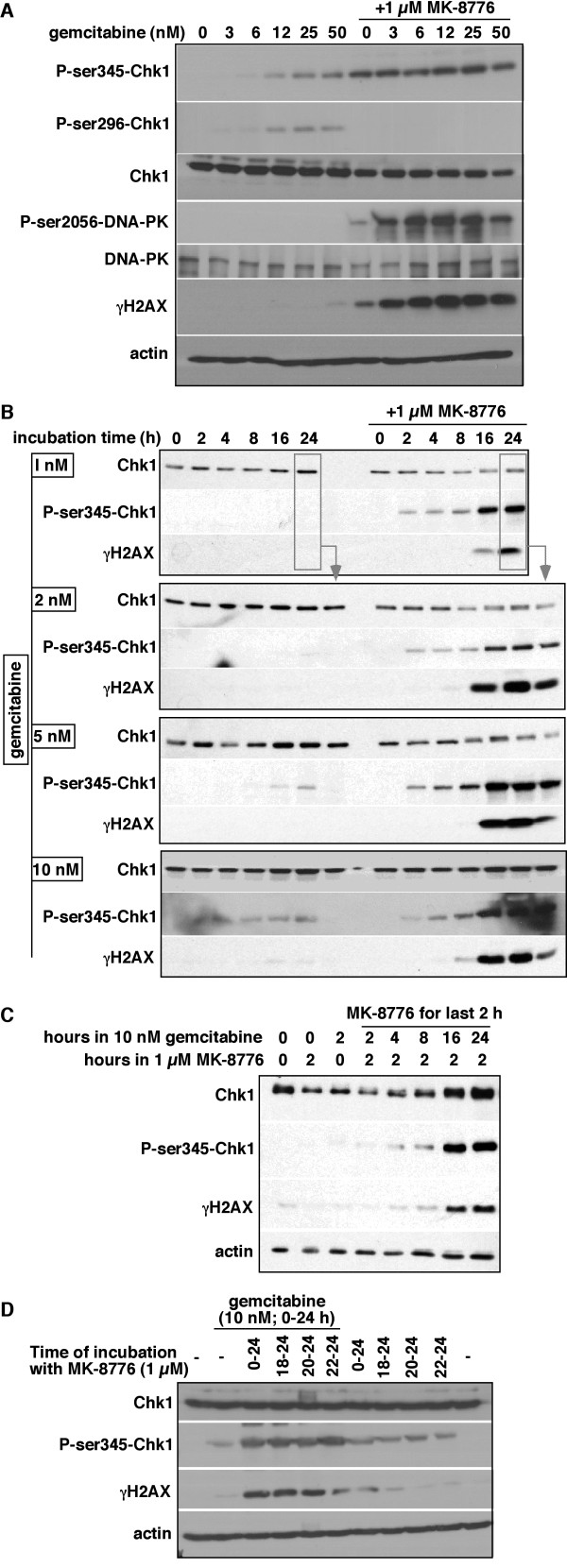
**Concentration and schedule dependence for the induction of DNA damage by gemcitabine plus MK-8776 in MDA-MB-231 cells. A**. Cells were incubated with the indicated concentration of gemcitabine for 24 h without, or concurrently with 1 μmol/L MK-8776. Cell lysates were analyzed by western blotting. **B**. Cells were incubated with 1–10 nmol/L gemcitabine for 0 – 24 h, without or with 1 μmol/L MK-8776. The 24-h sample incubated with 1 nmol/L gemcitabine was run on the other western blots to compare band intensities. **C**. Cells were incubated with or without gemcitabine for 0–24 h, and MK-8776 was added for the last 2 h. **D**. Cells were incubated with or without gemcitabine for 24 h, and MK-8776 was added concurrently or for the final 6, 4 or 2 h. Parallel cultures were incubated with MK-8776 alone. Cell lysates were analyzed by western blotting.

To further investigate the S phase arrest and whether it is caused primarily by inhibition of ribonucleotide reductase or by direct DNA damage, we asked whether these concentrations of gemcitabine activated Chk1. After a 24 h incubation of MDA-MB-231 cells with 50 nmol/L gemcitabine, there was marked phosphorylation of Chk1 at both ser345 and ser296 which suggests the presence of DNA damage, probably single-stranded regions in DNA as there was negligible phosphorylation either H2AX or DNA-protein kinase which should appear if there are DNA double-strand breaks (DSB) (Figure 2A). In contrast, no detectable phosphorylation of Chk1 was observed below 12 nmol/L suggesting little direct DNA damage occurs despite the fact that the cells arrest in early S phase at these concentrations.

Incubation of cells with MK-8776 alone for 24 h induced low level phosphorylation of ser345-Chk1. We have previously reported that this phosphorylation occurs prior to the detection of DNA damage as assessed by γH2AX [14], hence this is likely attributable to the inhibition of Chk1 preventing the normal feedback dephosphorylation by protein phosphatase 2A such that ongoing phosphorylation by ATR enhances phosphorylation of Chk1 [15]. When 1 μmol/L MK-8776 was combined with gemcitabine, even at the lowest concentrations tested, there was an increased phosphorylation of ser345-Chk1 but no phosphorylation of ser296-Chk1, an autophosphorylation site, consistent with inhibition of Chk1. There was also a dramatic increase in γH2AX and phospho-DNA-PK consistent with the collapse of replication forks. Contrary to a prior report [16], we did not see degradation of Chk1 by this combination, except marginally at the highest concentration, perhaps due to the much lower concentrations of gemcitabine used in the current study.

We next investigated the kinetics of phosphorylation of Chk1 and H2AX during incubation with 1–10 nmol/L gemcitabine, the concentrations around the IC50 concentrations of gemcitabine in combination with MK-8776 (Table 1). As anticipated from Figure 2A, there was negligible phosphorylation of Chk1 and H2AX in cells incubated with gemcitabine alone (Figure 2B). However, when the drugs were combined, high phosphorylation levels were observed, but this did not occur until 16 h. One possibility for this delay in the appearance of phospho-Chk1 and γH2AX is that the forks do not arrest rapidly. However, incubation of cells with 10 nmol/L gemcitabine caused complete suppression of DNA synthesis within 3 h (data not shown; but is also evident from the decrease in G_2_/M population after a 6-h incubation in Figure 1B).

### Impact of delaying addition of MK-8776 to gemcitabine-arrested cells

The above results suggest that, for the first 16 h of arrest, the replication forks do not depend on Chk1 for stability, but the stalled forks evolve with time to become more Chk1 dependent. To further test the time frame of Chk1 dependence, we added MK-8776 at different times after gemcitabine (Figure 2C). When added after 16 h, marked phosphorylation of Chk1 and H2AX occurred within 2 h consistent with the hypothesis that replication forks become more Chk1 dependent over time. To more directly compare the extent of DNA damage induced by these different schedules, we incubated cells with gemcitabine for 24 h, and added MK-8776 for the final 2, 4, 6 or 24 h (i.e., the latter being concurrent incubation). Incubation for just the final 4 h induced as much γH2AX as the concurrent incubation (Figure 2D). Hence, it is only necessary to add MK-8776 for a brief period once the replication forks have evolved to be Chk1 dependent.

Considering that the delayed addition of MK-8776 was as effective at inducing γH2AX, we assessed the impact of this schedule on cytotoxicity. In these experiments, gemcitabine was added for 24 h while MK-8776 was added for only the final 6 h (Table 1B). Marked sensitization was again observed, with only a slight decrease in extent of sensitization (~2 fold) compared to a 24 h concurrent treatment.

### Impact of MK-8776 on gemcitabine-induced homologous recombination

**Figure 3:**
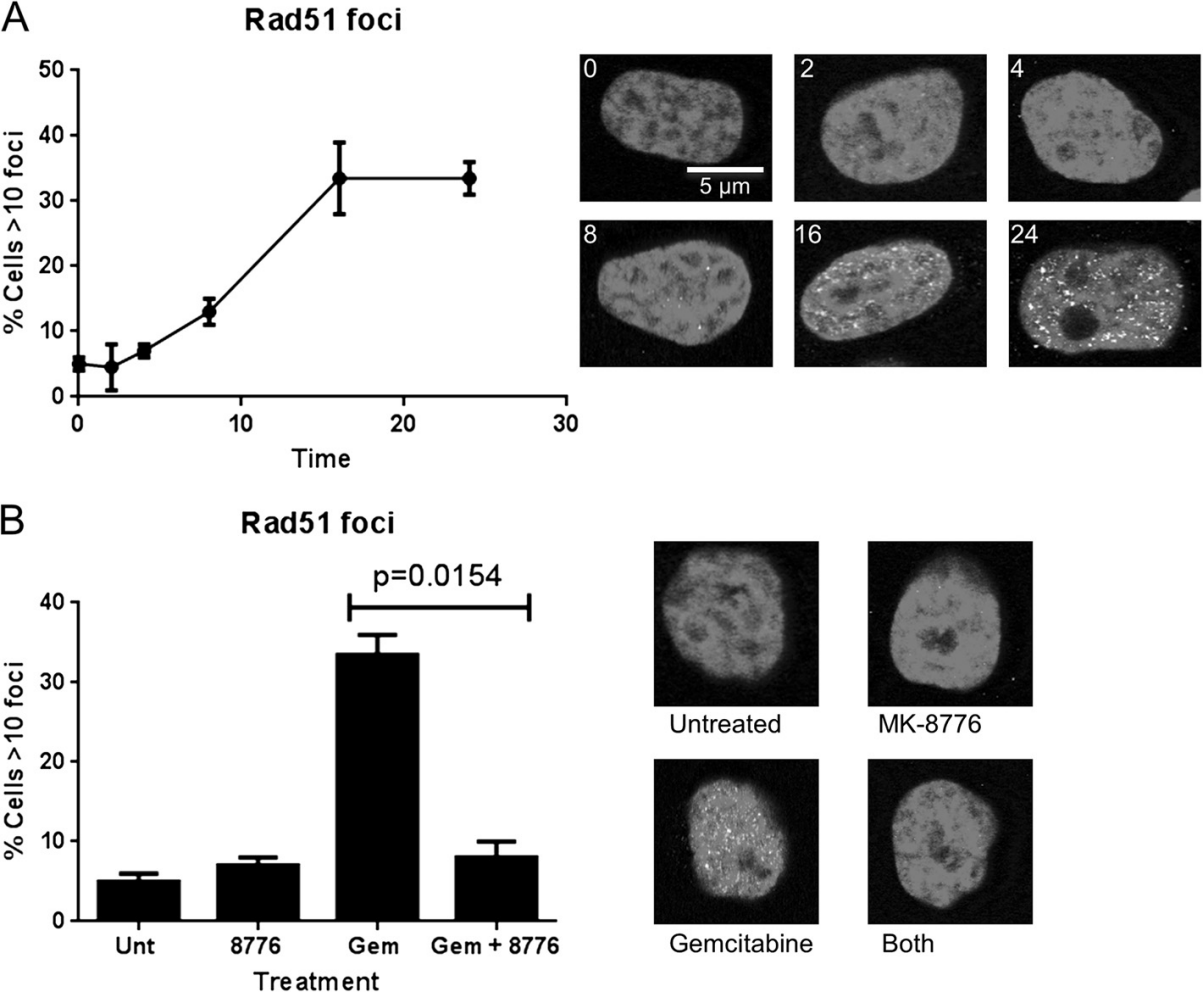
**Confocal imaging of RAD51 foci. A**. MDA-MB-231 cells were cultured on coverslips with 10 nmol/L gemcitabine for 0 – 24 h then stained for RAD51 foci. 100 cells were scored for each condition. Values reflect the mean and range of 2 independent experiments. **B**. Cells were untreated or incubated with either 1 μmol/L MK-8776 for 6 h, 10 nmol/L gemcitabine for 24 h, or 10 nmol/L gemcitabine 0–24 h with 1 μmol/L MK-8776 added for the last 6 h. Cells were scored as in A. Significance was calculated using an unpaired t-test.

Stalled replication forks provide a substrate for homologous recombination that can be visualized as the accumulation of nuclear RAD51 foci, and this step is dependent on Chk1 [16,17]. Gemcitabine has been shown to induce RAD51 foci after 24 h although the time of onset was not previously investigated [16]. To assess the kinetics of recombination following addition of gemcitabine, MDA-MB-231 cells were incubated with 10 nmol/L gemcitabine for 0–24 h, then fixed and stained for RAD51 foci. The number of cells with RAD51 foci began to increase at 8 h, but increased to about 35% of the cells by 16 and 24 h consistent with the percent of cells in S phase at the time of addition of gemcitabine (Figure 3A). It is worth noting that the cells still lack deoxyribonucleotides so the appearance of RAD51 foci does not reflect functional recombination but rather stalled recombination. This stalled recombination is eventually reversible once gemcitabine is removed as the cells were able to recover from this concentration of drug (Figure 1A).

When MK-8776 was added to gemcitabine-treated cells (i.e., at 18 h), RAD51 foci disappeared (Figure 3B). Hence, it appears that RAD51 protects the DNA from further damage, even though recombination has stalled, but when Chk1 is inhibited, Rad51 foci dissociate and replication forks collapse.

### Cell cycle perturbation and cytotoxicity induced by brief incubation with gemcitabine

The 6 h pulse of MK-8776 was selected above as it is consistent with the short half-life in patient plasma whereby concentrations above 1 μmol/L are only maintained for 6 h [8]. In a similar manner, gemcitabine is administered to patients as a bolus rather than a 24 h continuous incubation. While the parent drug has a short half-life in plasma, the activated nucleotides have a long intracellular half-life and consequently inhibit ribonucleotide reductase for a long period of time [18]. In addition, the inhibition of ribonucleotide reductase is irreversible further preventing recovery of the cells. However, the kinetics of cell cycle arrest following a bolus treatment have not been studied previously either *in vitro* or *in vivo*. This led us to investigate the consequences of a brief incubation with gemcitabine (nominally 6 h in these experiments). MDA-MB-231 cells were incubated with gemcitabine for 6 h, then the drug was removed and cell cycle perturbation assessed over the following 66 h (Figure 1B). In general, the results are similar to those observed following a 24 h continuous incubation with gemcitabine although about 4-fold higher drug concentration was required to induce arrest at mid or early S phase. The cells also recovered even at the highest concentration tested which was approximately the IC50 for a 6-h incubation with gemcitabine alone (Table 1C). However, when MK-8776 was added from 18–24 h, recovery was markedly reduced with cells remaining in S phase at the higher concentrations and an increase in sub-G1 population was apparent.

**Figure 4:**
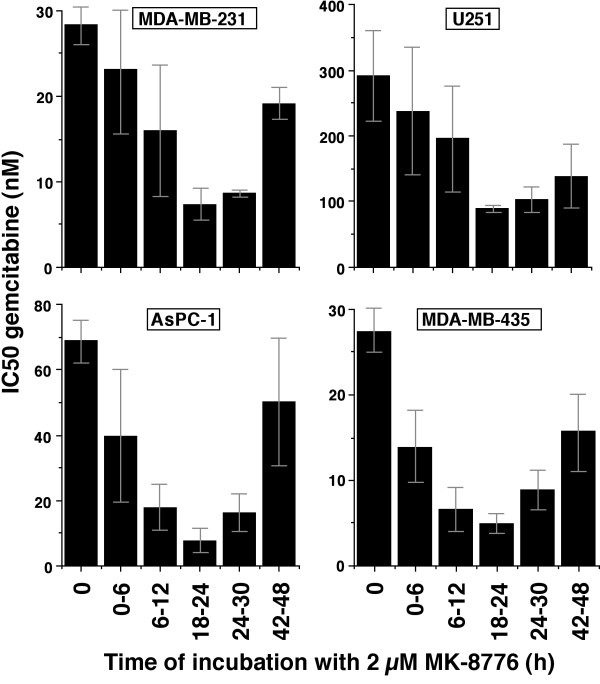
**Identification of the optimum schedule for combining gemcitabine and MK-8776.** The four indicated cell lines were incubated with gemcitabine for 6 h, and 2 μmol/L MK-8776 was added concurrently (column 2) or for a 6-h period at various times after removal of gemcitabine. After removal of drugs, cells were incubated for an additional 6–7 days and cell growth assayed based on DNA content. Experiments were performed in a 96 well format and results are expressed as 50% inhibition of growth of the culture. The values represent the mean and range for duplicate experiments. In addition, the mean and SEM of the values for additional experiments at 0 and 18–24 are presented in Table 1C.

To further investigate the optimal time of addition of MK-8776, we incubated cells with gemcitabine for 6 h, then added MK-8776 either concurrently or for 6-h periods at various times after removal of gemcitabine (Figure 4). While concurrent incubation decreased the IC_50_ for gemcitabine by almost 50%, the greatest sensitization was observed when MK-8776 was administered from 18–24 h (i.e., 12–18 h after removal of gemcitabine). This experiment was extended to three other cell lines, and all showed the same result whereby addition of MK-8776 from 18–24 h had the greatest impact on the IC_50_ for gemcitabine.

The impact of this schedule (gemcitabine 0–6 h and MK-8776 18–24 h) was assessed in additional cell lines (Table 1C). The brief incubation with gemcitabine was generally 2–8 fold less cytotoxic than the 24 h continuous incubation. However, the addition of 2 μmol/L MK-8776 still induced 2–10 fold sensitization to gemcitabine.

### Cell cycle perturbation induced by gemcitabine *in vivo*

These experiments were extended to xenograft models to determine the extent of cell cycle arrest following administration of gemcitabine. Ki67 is often used as a marker of proliferation but cells at any phase of the cell cycle, except Go, are positive for this antigen. In contrast, only cells in S and G2 express geminin [19]. Accordingly, the ratio of geminin/Ki67 reflects the proportion of cells in the cell cycle (Ki67 positive) that are in S or G2 (geminin positive) at the time of harvest. This ratio (expressed here as a percentage) corrects for large differences in Ki67-positive cells throughout a tumor which can result from hypoxia or limited nutrient supply.

In preliminary studies, we found that some tumor models were not very amenable to this analysis. For example, the MDA-MB-231 cells exhibited a very narrow rim of proliferating cells surrounding a large Ki67-negative center. Several other tumors including U87 glioma expressed very low levels of geminin. However, AsPC-1 and MiaPaCa-2 pancreas xenografts showed good distribution of both antigens throughout the tumor and were therefore used in these studies. These cells were first analyzed *in vitro* to confirm their cell cycle perturbation following gemcitabine. Both cell lines showed S phase arrest and recovery following a 6-h incubation with gemcitabine that was comparable to that seen in MDA-MB-231 cells but at 4–8 fold higher concentration (Additional file 1: Figure S1 and Figure S2). Addition of MK-8776 from 18 – 24 h (12 h after removal of gemcitabine) caused sustained arrest of the cells that did not resolve by 72 h (similar to MDA-MB-231 cells).

**Figure 5:**
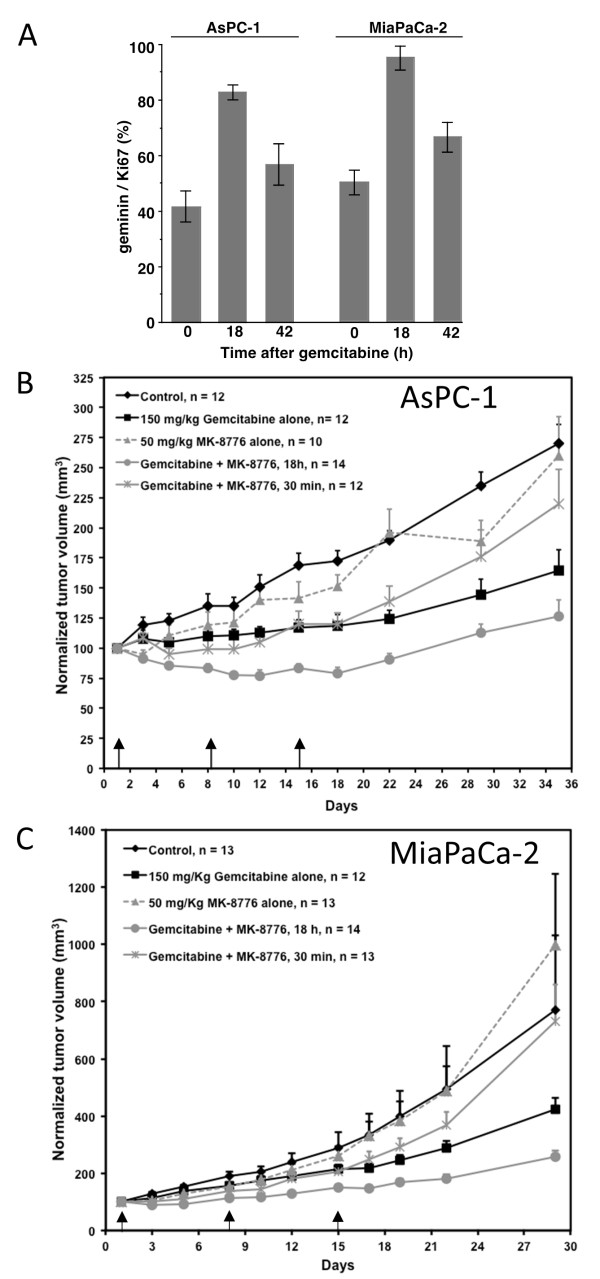
**Impact of gemcitabine and MK-8776 on two pancreas tumor xenografts. A**. Mice were administered 150 mg/kg gemcitabine and tumors harvested at 18 h and 42 h. Serial sections from the tumors were stained for Ki67 and geminin and the ratio of geminin/Ki67 expressed as a percentage. Results represent the mean and SEM for at least 2 sections from 2–4 mice. **B**. Mice bearing AsPC-1 tumors were administered 150 mg/kg gemcitabine on days 1, 8, 15 (arrows); or 50 mg/kg MK-8776; or the combination of these two drugs with MK-8776 given either 30 min or 18 h after gemcitabine. Data are expressed as mean and SEM for each time point. “n” represents the number of mice in each group. After day 5, all gemcitabine-treated groups were significantly different from untreated mice (p < 0.05). Treatment with gemcitabine followed by MK-8776 after 30 min was not significantly different than gemcitabine alone. Treatment with gemcitabine followed by MK-8776 after 18 hours was significantly different from gemcitabine alone or when combined with MK-8776 after 30 minutes (p < 0.05). **C**. Mice bearing MiaPaCa-2 tumors were treated as in **B**. After 12 days, treatment with gemcitabine followed by MK-8776 after 18 hours was significantly different from either gemcitabine alone or when combined with MK-8776 after 30 minutes (p < 0.05).

Mice bearing these pancreas xenografts were administered 150 mg/kg gemcitabine and tumors harvested after either 18 or 42 h. The tumors were then stained for Ki67 and geminin. In untreated tumors, Ki67-positive cells were distributed through much of the tumor, but in those areas where it was most abundant, it still only represented about half of the cells (Additional file 1: Figure S3). Serial sections of the slides showed geminin had a similar distribution, but with a lower frequency (40-50% of Ki67-positive cells; Figure 5A). Treatment with gemcitabine increased the frequency of geminin-positive cells to 83% at 18 h in AsPC-1 xenografts and 95% in MiaPaCa2, but the cells began to recover by 42 h. These results show that gemcitabine induces a large but transient arrest of the cells in S phase at 18 h.

### Impact of gemcitabine plus MK-8776 on tumor growth delay

The two pancreas xenografts were also used to assess the response to gemcitabine plus MK-8776. Tumor-bearing mice were administered gemcitabine alone, MK-8776 alone or in combination using two different schedules: MK-8776 was administered either 30 min or 18 h after gemcitabine. Mice were treated each week for three weeks (days 1, 8 and 15) and tumor volume and mouse weight recorded. Untreated AsPC-1 tumors doubled in volume over approximately 22 days whereas MiaPaCa-2 doubled in approximately 10 days (Figure 5B and C). Administration of MK-8776 alone was not significantly different than control in either model. Gemcitabine treatment caused a significant decrease in growth rate, but did not cause any tumor regression. MK-8776 administered 30 min after gemcitabine was not significantly different than gemcitabine alone. In contrast, when MK-8776 was administered 18 h after gemcitabine, the tumor growth rate was significantly slower than all other groups, and in AsPC-1, partial tumor regression was observed (about 25% by day 10); partial recovery occurred after the third treatment, although the tumor size remained significantly less than all other treatment groups throughout the experiments. No obvious toxicity to the mice was observed and there was no significant difference in weight between any of the groups, albeit a slight (5%) loss of weight appeared to occur transiently following administration of MK-8776 on all schedules (data not shown). This experiment confirms that delaying administration of MK-8776 for 18 h after gemcitabine is well tolerated and has the greater therapeutic potential.

## Discussion

Chk1 participates in multiple functions in a cell [3]. It was originally recognized as a mediator of the DNA damage response, preventing cell cycle progression so that cells could repair DNA damage. The underlying mechanism involves Chk1-mediated inhibition of CDC25, thereby preventing activation of CDK1 and 2. Inhibition of Chk1 leads to activation of CDK1/2, cell cycle progression and aberrant mitosis. Recently, it has been recognized that some cell lines are hypersensitive to brief inhibition of Chk1 alone, with γH2AX foci and/or DNA double-strand breaks appearing within 6 h [6,14,20]. This damage occurs only in S phase cells and is also mediated by activation of CDK2. In addition, Chk1 is now recognized as having additional roles in replication fork stability, replication origin firing and homologous recombination, and it is the latter of these roles that appears important for the efficacy of the combination of gemcitabine with MK-8776. Mechanistically, homologous recombination results when Chk1 phosphorylates the C-terminal domain of BRCA2 which then interacts with and recruits RAD51 to single-stranded DNA. In addition Chk1 can directly phosphorylate RAD51 and this is also required for recruitment of RAD51 to single-stranded DNA [17,21]. Our results demonstrate that inhibition of Chk1 can also result in dissociation of RAD51 from DNA which we suggest is due to the dynamic status of regressed replication forks which likely shorten or grow in length continuously and thereby displace RAD51.

These different functions of Chk1 can explain why Chk1 inhibitors exhibit variable efficacy in sensitizing cells to DNA damaging agents. Our previous experiments involved incubation of cells with the topoisomerase I inhibitor SN38 [4,6]. Replication forks collide with the inhibited topoisomerase complex creating DNA breaks that rapidly activate Chk1 and prevent cell cycle progression. Yet while inhibition of Chk1 induced cell cycle progression, it had little impact on overall cytotoxicity because lethal breaks were already induced by SN38 alone. In contrast, when gemcitabine or hydroxyurea inhibit ribonucleotide reductase, replication stalls rapidly and independently of Chk1. Indeed, we previously demonstrated that hydroxyurea can arrest DNA replication without activating Chk1, and this observation is reiterated here at low concentrations of gemcitabine [6]. Upon removal of gemcitabine, these arrested cells are able to recover. However, inhibition of Chk1 rapidly induces collapse of replication forks, and this is new DNA damage that dramatically enhances cell killing. Other investigators have observed activation of Chk1 upon incubation with either hydroxyurea or gemcitabine, but in general those experiments involved higher concentrations of each drug that exceed those needed to arrest the cells [22-24]. We have observed slight activation of Chk1 when western blots are over-exposed, but this level of phosphorylation is far lower than observed after replication forks have collapsed as a consequence of Chk1 inhibition. Similar observations were made in a study of gemcitabine alone which showed phosphorylation of Chk1, but a subsequent paper also showed this to be negligible compared to that induced by concurrent inhibition of Chk1 [25,26]. In the case of cells incubated with gemcitabine alone, we question whether the low level activation of Chk1 is due to incorporation of gemcitabine into DNA and the chain termination that then occurs rather than to the inhibition of ribonucleotide reductase.

Here, we show that MK-8776 markedly sensitizes multiple cell lines to gemcitabine. In further dissecting the mechanism, we noted that γH2AX did not appear until about 16 h of co-treatment. We therefore delayed the addition of MK-8776 and demonstrated that, when added for the final 4 h of a 24-h incubation of gemcitabine, it induced as much γH2AX signal as it did when incubated concurrently with gemcitabine for the entire 24 h. Our results demonstrate that stalled replication forks evolve with time to become more Chk1 dependent, and this correlates with a delay in loading of Rad51 onto DNA. When Chk1 was inhibited, these Rad51 foci disappeared and very strong γH2AX signal was observed. Evolution of stalled replication forks and delayed appearance of RAD51 foci have previously been observed during incubation with hydroxyurea, but it was concluded that RAD51-dependent recombination occurred in response to collapsed replication forks [27]. Here we observed very few γH2AX-positive foci prior to recombination, but a dramatic increase once RAD51 loading was prevented by inhibiting Chk1. This implies that the appearance of γH2AX is a consequence of inhibiting recombination and not the stimulus for recombination. That inhibition of recombination is important for the observed sensitization is also suggested by the TK10 cells which were sensitive to gemcitabine alone, and were not further sensitized by MK-8776. This cell line has been reported to have a defect in recombination which would explain this observation [28].

The requirement for only a brief incubation with MK-8776 to enhance cytotoxicity is an important observation given that, in clinical trials, the plasma concentration of MK-8776 was shown to exceed 1 μmol/L for only about 6 h [8]. MK-8776 dissociates rapidly from Chk1 when the drug is removed (data not shown), so it is unlikely that Chk1 will remain inhibited significantly beyond 6 h.

We extended these experiments to more closely reflect the clinical situation by incubating cells briefly with gemcitabine, and then permitting the cells to recover. Because ribonucleotide reductase remains inhibited for a long time, it took several days for the cells to recover; the rate of recovery depended on the concentration of gemcitabine. Cells in G_1_ also progressed into S phase during this time, so the number of cells potentially susceptible to Chk1 inhibition continued to increase. Hence there are two reasons why delayed addition of MK-8776 can enhance sensitivity to gemcitabine: first, there is an increased number of cells arrested in S phase, and second, the arrested cells have been given adequate time to become Chk1 dependent (i.e., to initiate recombination). The current experiments indicated that addition of MK-8776 at 18 h provided the greatest decrease in IC50 for gemcitabine in four cell lines (Figure 4). However, these experiments only reflect growth inhibition, and the S phase arrest at these low concentrations was very transient. Higher concentrations of gemcitabine induce a longer arrest with more cells accumulating in S phase. Consequently, it is possible that later addition of MK-8776 may have improved cell killing as the cells newly arrested in S phase at 18 h may not yet have become Chk1 dependent.

To more directly assess the relevance of these *in vitro* observations, we assessed the S/G_2_ phase arrest that occurred in two different tumor models *in vivo*. This was quantified as the ratio of geminin-positive to Ki67-positive cells. Eighteen hours after administration of 150 mg/kg gemcitabine, there was a marked increase in geminin-positive cells suggesting that up to 83-95% of the Ki67-positive cells were in S or G_2_ phase. By 42 h, this percentage had partially reverted to the starting value reflecting recovery of the cells. This dose of gemcitabine is considered equivalent to a dose of 450 mg/m^2^ in patients, which is about half the standard dose administered (1000 mg/m^2^). We are currently performing a clinical trial to assess the S/G_2_ phase arrest that occurs in patients receiving gemcitabine as a guide for subsequent administration of a Chk1 inhibitor.

Finally, we assessed the impact of schedule on the response of human tumor xenografts to the combination of gemcitabine and MK-8776. The results clearly demonstrated that administration of MK-8776 18 h after gemcitabine, but not 30 min after, caused significant decrease in tumor growth compared to gemcitabine alone, consistent with the observations made *in vitro*. This conclusion held in two different tumor models. The pharmacokinetics of MK-8776 in mice is currently being assessed, and we believe it may be possible to increase the length of exposure of tumors to drug and thereby further enhance the therapeutic response.

The clinical development of Chk1 inhibitors has taken many years. The first candidate, UCN-01, was a broad kinase inhibitor but had unfavorable pharmacokinetic properties [29,30]. Three subsequent Chk1 inhibitors that entered clinical trial, AZD7762, XL9844 and PF-00477736, have been discontinued; whether this is due to mechanism-based toxicity or off-target effects remains to be determined (the latter drug was reportedly terminated for business reasons rather than concerns for safety or efficacy; http://www.clinicaltrials.gov). Clinical trials are currently ongoing with LY2606318, LY2606368 and GDC-0425. In most cases, these inhibitors are being studied in combination with gemcitabine or, in one case, pemetrexed [31]. One issue with all these drugs is that they inhibit several other targets, and in most cases this includes Chk2, although the published information is limited. Indeed, there are currently no publications reflecting the preclinical development of these other agents with which we can compare our current results.

MK-8776 may have an advantage over other Chk1 inhibitors in being much more selective for Chk1 and additionally, it does not inhibit Chk2 [7]. MK-8776 has completed Phase I clinical trials in combination with gemcitabine although the schedule was based on a 30 min interval between the two drugs. The results of a second Phase I clinical trial in combination with cytarabine has just been reported [8]. In this case a different schedule was used: cytarabine was administered as a 72 h infusion with MK-8776 given on day 2 and 3 [8]. The schedule with other Chk1 inhibitors could vary depending upon the time frame over which it can inhibit Chk1, and the DNA damaging agent with which it is combined. For example, LY2603618 has recently been shown to have a plasma half-life of 5 – 25 h, though whether this drug remains bioavailable throughout this time frame is unknown [31]. Our results provide justification for a schedule of administration whereby gemcitabine is administered 18 h prior to MK-8776, and this justification should apply to clinical trials of gemcitabine with any other Chk1 inhibitor.

## Conclusions

Chk1 inhibitors have shown great promise in preclinical experiments, particularly when used to sensitize tumors to antimetabolites such as gemcitabine. However, prior experiments have not defined the best schedule for administration of these two drugs. We have identified two reasons that justify delaying administration of MK-8776 until 18 h after gemcitabine: first, there is an increased number of cells arrested in S phase; and second, the arrested cells become increasingly dependent on Chk1 over time due to their reliance on homologous recombination. Consequently, the delayed administration of MK-8776 provides greater tumor growth delay in xenograft models. These results have important implications for the design of clinical trials of this drug combination.

## Competing interests

The authors declare that they have no competing of interests.

## Authors’ contributions

AE designed the overall study. NK designed the in vivo experiments. RM performed the majority of the in vitro experiments with help from RT, IC and AE. HH and NK performed the in vivo experiments. RM and AE wrote the manuscript which was then reviewed and approved by all other authors.

## Pre-publication history

The pre-publication history for this paper can be accessed here:

http://www.biomedcentral.com/1471-2407/13/604/prepub

## Supplementary Material

Additional file 1: **Montano et al. Sensitization of human cancer cells to gemcitabine by the Chk1 inhibitor MK-8776: cell cycle perturbation and impact of administration schedule *in vitro *** **and *in Vivo. *** **Figure S1.** Impact of gemcitabine and MK-8776 on cell cycle perturbation of AsPC-1 cells. Cells were incubated with 0 – 200 nM gemcitabine for 6 h, then the drug was removed and cells incubated for up to 72 h (left). One set of cells (right) were also incubated with 1 μM MK-8776 from 18-24 h. **Figure S2.** Impact of gemcitabine and MK-8776 on cell cycle perturbation of MiaPaCa-2 cells. Cells were incubated with 0 – 200 nM gemcitabine for 6 h, then the drug was removed and cells incubated for up to 72 h (left). One set of cells (right) were also incubated with 1 μM MK-8776 from 18-24 h. **Figure S3.** Impact of gemcitabine on cell cycle perturbation in AsPC-1 (top) and MiaPaCa-2 (bottom) tumor xenografts. Tumors from untreated mice, or mice administered 150 mg/kg gemcitabine were harvested at 18 h. Serial sections from the tumors were stained for Ki67 and geminin. Representative immunohistochemistry is shown. Quantification is presented in Figure 5. Click here for file
