# Supplementary material for: Sensitization of human cancer cells to gemcitabine by the Chk1 inhibitor MK-8776: cell cycle perturbation and impact of administration schedule in vitro and in vivo
Source: BMC Cancer. 2013 Dec 21;13:604. doi: 10.1186/1471-2407-13-604 (PMC3878047; doi:10.1186/1471-2407-13-604)

Supplemental Information

Montano et al, Sensitization of human cancer cells to gemcitabine by the Chk1 inhibitor MK-8776: cell cycle perturbation and impact of administration schedule *in vitro* and *in vivo*

Figure S1. Impact of gemcitabine and MK-8776 on cell cycle perturbation of AsPC-1 cells. Cells were incubated with 0 – 200 nM gemcitabine for 6 h, then the drug was removed and cells incubated for up to 72 h (left). One set of cells (right) were also incubated with 1 μM MK-8776 from 18-24 h.

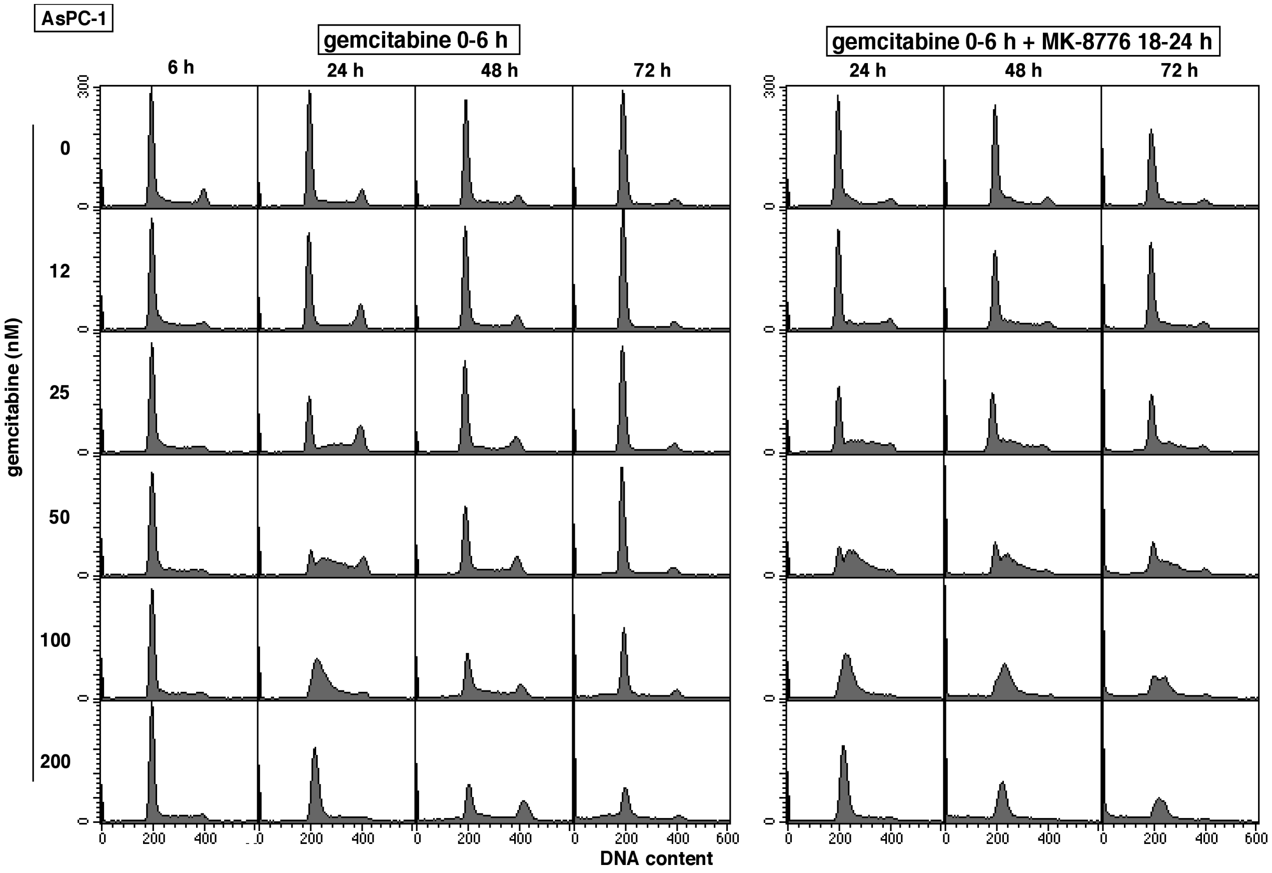

Figure S1

Figure S2. Impact of gemcitabine and MK-8776 on cell cycle perturbation of MiaPaCa-2 cells. Cells were incubated with 0 – 200 nM gemcitabine for 6 h, then the drug was removed and cells incubated for up to 72 h (left). One set of cells (right) were also incubated with 1 μM MK-8776 from 18-24 h.

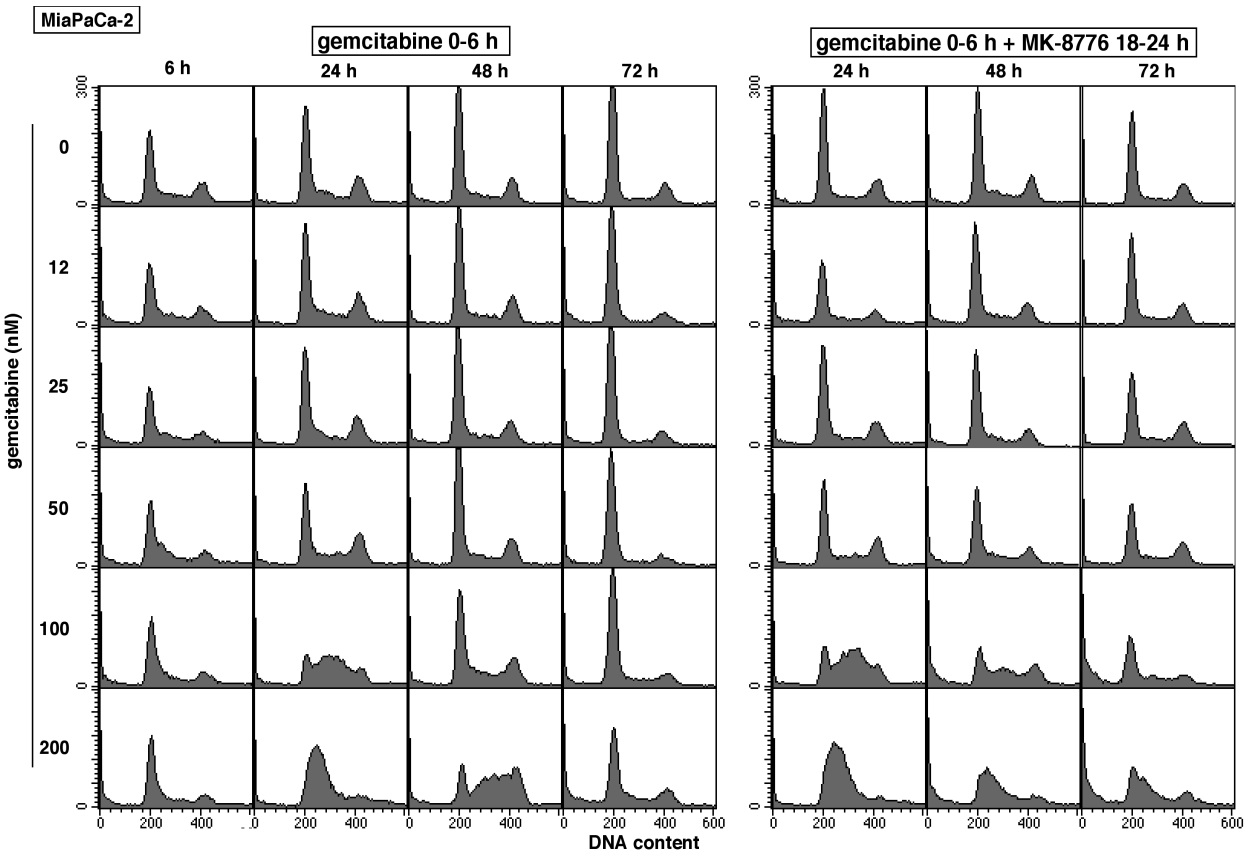

Figure S3. Impact of gemcitabine on cell cycle perturbation in AsPC-1 (top) and MiaPaCa-2 (bottom) tumor xenografts. Tumors from untreated mice, or mice administered 150 mg/m^2 gemcitabine were harvested at 18 h. Serial sections from the tumors were stained for Ki67 and geminin. Representative immunohistochemistry is shown. Quantification is presented in Figure 5.

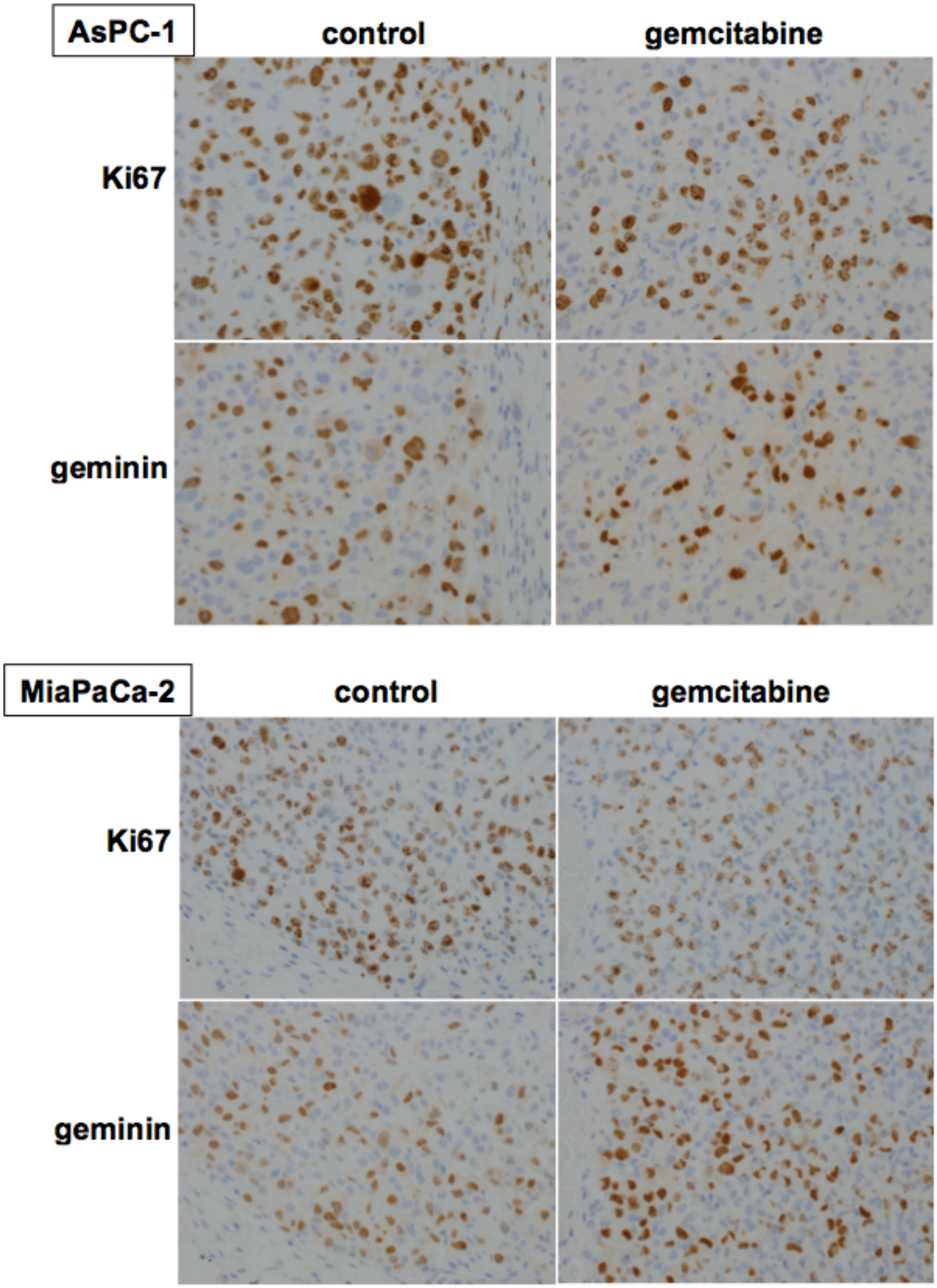

Supplement: Additional file 1: — Montano et al. Sensitization of human cancer cells to gemcitabine by the Chk1 inhibitor MK-8776: cell cycle perturbation and impact of administration schedule in vitro and in Vivo. Figure S1. Impact of gemcitabine and MK-8776 on cell cycle perturbation of AsPC-1 cells. Cells were incubated with 0 – 200 nM gemcitabine for 6 h, then the drug was removed and cells incubated for up to 72 h (left). One set of cells (right) were also incubated with 1 μM MK-8776 from 18-24 h. Figure S2. Impact of gemcitabine and MK-8776 on cell cycle perturbation of MiaPaCa-2 cells. Cells were incubated with 0 – 200 nM gemcitabine for 6 h, then the drug was removed and cells incubated for up to 72 h (left). One set of cells (right) were also incubated with 1 μM MK-8776 from 18-24 h. Figure S3. Impact of gemcitabine on cell cycle perturbation in AsPC-1 (top) and MiaPaCa-2 (bottom) tumor xenografts. Tumors from untreated mice, or mice administered 150 mg/kg gemcitabine were harvested at 18 h. Serial sections from the tumors were stained for Ki67 and geminin. Representative immunohistochemistry is shown. Quantification is presented in Figure 5. [file 1471-2407-13-604-S1.pdf]
